# Anti-Parkinson Activity of Petroleum Ether Extract of* Ficus religiosa* (L.) Leaves

**DOI:** 10.1155/2016/9436106

**Published:** 2016-01-17

**Authors:** Jitendra O. Bhangale, Sanjeev R. Acharya

**Affiliations:** Institute of Pharmacy, Nirma University, Ahmedabad, Gujarat 382 481, India

## Abstract

In the present study, we evaluated anti-Parkinson's activity of petroleum ether extract of* Ficus religiosa* (PEFRE) leaves in haloperidol and 6 hydroxydopamine (6-OHDA) induced experimental animal models. In this study, effects of* Ficus religiosa* (100, 200, and 400 mg/kg, p.o.) were studied using in vivo behavioral parameters like catalepsy, muscle rigidity, and locomotor activity and its effects on neurochemical parameters (MDA, CAT, SOD, and GSH) in rats. The experiment was designed by giving haloperidol to induce catalepsy and 6-OHDA to induce Parkinson's disease-like symptoms. The increased cataleptic scores (induced by haloperidol) were significantly (*p* < 0.001) found to be reduced, with the PEFRE at a dose of 200 and 400 mg/kg (p.o.). 6-OHDA significantly induced motor dysfunction (muscle rigidity and hypolocomotion). 6-OHDA administration showed significant increase in lipid peroxidation level and depleted superoxide dismutase, catalase, and reduced glutathione level. Daily administration of PEFRE (400 mg/kg) significantly improved motor performance and also significantly attenuated oxidative damage. Thus, the study proved that* Ficus religiosa* treatment significantly attenuated the motor defects and also protected the brain from oxidative stress.

## 1. Introduction

Parkinson's disease (PD) is caused by the gradual and selective loss of dopaminergic neurons in the substantia nigra pars compacta (SNpc) [1, 2]. PD produces bradykinesia, muscular rigidity, rest tremor, and loss of postural balance along with some secondary manifestations like dementia, sialorrhoea [3], soft speech, and difficulty in swallowing due to uncoordinated movements of mouth and throat [4]. PD occurs due to inhibition of mitochondrial complex-1 [5, 6], different mechanisms of cell damage like excitotoxicity, calcium homeostasis, inflammation, apoptosis, distressed energy metabolism, and protein aggregation [7], and interaction between genetic and environmental factors [8].

Oxidative stress interferes with dopamine metabolism leading to Parkinson's disease. This oxidative damage leads to formation of reactive oxygen species (ROS) leading to neuronal death [9, 10]. This was evidenced by reduced level of endogenous antioxidant compounds. These findings introduced the requirement of using antioxidants as a therapeutic intervention in PD in addition to other protective agents.

The current available drug treatments for PD possess various side effects. Therefore, herbal therapies should be considered as alternative/complementary medicines for therapeutic approach.

Since ancient times, plants have been an ideal source of medicine. Plants have played a noteworthy role in maintaining human health and improving the quality of life for thousands of years and have served humans as well, as valuable components of medicines, seasonings, beverages, cosmetics, and dyes. In modern times, focus on plant research has increased all over the world and a large body of evidence has been collected to demonstrate immense potential of medicinal plants used in various traditional systems.* Ficus religiosa* Linn. (Moraceae) commonly known as “Pimpala” or “Pipal” tree is a large widely deciduous tree, heart-shaped without aerial roots from the branches, with spreading branches and grey bark [11–13]. The tree is held sacred by Hindus and Buddhists. In India it is known by several vernacular names, the most commonly used ones being Asvatthah (Sanskrit), Sacred fig (Bengali), Peepal (Hindi), Arayal (Malayalam), Ravi (Telugu), and Arasu (Tamil). Leaves contain campesterol, stigmasterol, isofucosterol, *α*-amyrin, lupeol, tannic acid, arginine, serine, aspartic acid, glycine, threonine, alanine, proline, tryptophan, tyrosine, methionine, valine, isoleucine, leucine, n-nonacosane, n-hentricontane, hexacosanol, and n-octacosane [14, 15].* Ficus religiosa* has been used in traditional medicine for a wide range of ailments. Its bark, fruits, leaves, roots, latex, and seeds are medicinally used in different forms, sometimes in combination with other herbs [16]. The whole parts of the plant exhibit wide spectrum of activities such as anticancer, antioxidant, antidiabetic, antimicrobial, anticonvulsant, anthelmintic, antiulcer, antiasthmatic, and antiamnesic activities. Bark of the plant has been used as astringent, cooling, aphrodisiac, and antibacterial against* Staphylococcus aureus* and* Escherichia coli*, gonorrhea, diarrhea, dysentery, hemorrhoids, and gastrohelcosis, as anti-inflammatory, and for burns. The leaves of the plant have been used for hemoptysis, epistaxis, hematuria, menorrhagia, blood dysentery, and skin diseases. Leaf juice has been used for asthma, cough, sexual disorders, diarrhea, hematuria, toothache, migraine, eye troubles, gastric problems, and scabies. Fruits of the plant were used in asthma and as laxative and digestive. Seeds of the plant were used as refrigerant and laxative and latex was used in neuralgia, inflammations, and hemorrhages [17]. As* F. religiosa* has been used traditionally in the treatment of neurodegenerative disorders (including Parkinson's disease) and has also been reported to possess antioxidant activity, this plant may prove to be effective in the remedy of PD. Hence* F. religiosa* was evaluated for its anti-Parkinson's effect using neurotoxin induced Parkinson's model in rats.

## 2. Materials and Methods

### 2.1. Collection of Plant Material

Fresh leaves of* Ficus religiosa* were collected from local area of Ahmedabad district, Gujarat, India, during July–September. This plant was identified and authenticated by Dr. A. Benniamin, Scientist D, Botanical Survey of India, Pune. Voucher specimens number BSI/WC/Tech./2015/JOB-1 have been kept in Botanical Survey of India, Pune, Maharashtra, India.

### 2.2. Animals

Adult male Wistar rats, weighing 180–220 g, and albino mice of either sex weighing 25–30 g were used and acclimatized to laboratory condition for one week. All animals were housed in well-ventilated polypropylene cages at 12 : 12 h light/dark schedule at 25 ± 2°C and 55–65% RH. The rats were fed with commercial pelleted rats chow and water* ad libitum* as a standard diet. Institutional Animal Ethics Committee approved the experimental protocol in accordance with the Committee for the Purpose of Control and Supervision of Experiments on Animals (CPCSEA).

### 2.3. Preparation of Leaf Extract

The leaves were collected and dried in shade and ground. Coarsely powdered plant material (1000 g) was weighed and extracted with 5 lit of solvents like petroleum ether (60–80°C), ethyl acetate, and ethanol by successive extraction in a Soxhlet apparatus for 72 h. After each extraction, the solvent was distilled off and concentrated extract was transferred to previously weighed petri dish and evaporated to dryness at room temperature (45–50°C) to obtain dried extracts. The dried extract was weighed and the percentage yield of the extracts was calculated as follows:

(1) %  of extractive yieldw/w=Weight of dried extractWeight of dried leaves powder×100.

The yield of petroleum ether, ethyl acetate, and ethanol extract was 18.2, 10.6, and 26.8% (w/w), respectively.

### 2.4. Preliminary Phytochemical Studies

Preliminary qualitative phytochemical screening was done for the presence of different group of chemicals, that is, alkaloids, flavonoids, saponins, tannins, sterols, carbohydrates, and glycosides, as described by Harborne [18].

#### 2.4.1. Test for Tannins and Phenols

5 mL of extract was added to 2 mL of 5% of alcoholic FeCl_3_ solution. Blue-Black precipitate indicated the presence of tannins and phenols.

#### 2.4.2. Test for Alkaloids

To the dry extract (10–20 mg), dilute hydrochloric acid (1-2 mL) was added, shaken well, and filtered. With filtrate, the following tests were performed.

*(1) Mayer's Test*. To 2-3 mL of filtrate, 2-3 drops of Mayer's reagent were added. Appearance of precipitate indicated presence of alkaloids.

*(2) Wagner's Test*. To 2-3 mL of filtrate, Wagner's (3–5 drops) reagent was added. Appearance of reddish-brown precipitate indicated presence of alkaloids.

*(3) Hager's Test*. To 2-3 mL of filtrate, 4-5 drops of Hager's reagent were added. Appearance of yellow precipitate indicated presence of alkaloids.

*(4) Dragendorff's Test*. To 2-3 mL of filtrate, 4-5 drops of Dragendorff's reagent were added. Appearance of orange-brown precipitate indicated presence of alkaloids.

#### 2.4.3. Test for Saponins

About 1 g of dried powdered sample was boiled with 10 mL distilled water. Frothing persistence indicated the presence of saponins.

#### 2.4.4. Test for Terpenoids

5 mL of extract was mixed with 2 mL of chloroform and few drops of concentrated H_2_SO_4_ were carefully added to form a layer. Red ring indicated that the terpenoids are present.

#### 2.4.5. Test for Steroids (Liebermann-Burchard Reaction)

5 mL of extract was mixed with 10 mL CHCl_3_ and 1 mL acetic anhydride and few drops of concentrated H_2_SO_4_ were added. Green ring indicated the presence of steroids.

#### 2.4.6. Test for Flavonoids (Shinoda Test)

To dry extract (10–20 mg), 5 mL of ethanol (95%), 2-3 drops of hydrochloric acid, and 0.5 g magnesium turnings were added. Change of color of solution to pink indicated presence of flavonoids.

#### 2.4.7. Test for Carbohydrates (Molisch's Test)

Few drops (2-3) of *α*-naphthol solution in alcohol were added to 2-3 mL of solution of extract and shaken for few minutes and then 0.5 mL of conc. sulfuric acid was added from the side of test tube. The formation of violet ring at the junction of two solutions indicated presence of carbohydrates.

#### 2.4.8. Test for Glycosides

*(1) Legal's Test*. To the extract, 1 mL of pyridine and 1 mL of sodium nitroprusside were added. Change in color to pink or red indicated presence of cardiac glycosides.

*(2) Keller-Kiliani Test*. Glacial acetic acid (3–5 drops), one drop of 5% ferric chloride, and concentrated sulfuric acid was added to the test tube containing 2 mL of solution of extract. Appearance of reddish-brown color at the junction of two layers and bluish green in the upper layer indicated presence of cardiac glycosides.

*(3) Borntrager's Test*. Dilute sulfuric acid was added to 2 mL of solution of extract, boiled for few minutes, and filtered. To the filtrate, 2 mL of benzene or chloroform was added and shaken well. The organic layer was separated and ammonia was added. The change in color of ammoniacal layer to pink red indicated presence of anthraquinone glycosides.

#### 2.4.9. Test for Phlobatannins

About 2 mL of extract was boiled with 2 mL 1% HCl. Deposition of red color indicated the presence of phlobatannins.

#### 2.4.10. Test for Amino Acid (Ninhydrin Test)

5 to 6 drops of Ninhydrin reagent were added in 5 mL of extract and heated over boiling water bath for 5 min. Purple coloration indicated the presence of amino acid.

#### 2.4.11. Test for Proteins (Biuret Test)

5-6 drops of 5% NaOH and 5–7 drops of 1% CuSO_4_ were added in 2 mL of extract. Violet color indicated the presence of protein.

### 2.5. Acute Oral Toxicity of the Extract

The mice were divided into 5 groups of 10 animals each. The mice were fasted for 6 h and had access to only water* ad libitum* before experimental study. Group I received only vehicle (distilled water). Groups II, III, IV, and V received different doses of pet. ether extract of* F. religiosa* (PEFRE), that is, 1000, 2000, 3000, and 4000 mg/kg, respectively. All the doses and vehicle were administered orally. The animals were observed for 72 h for mortality [19].

### 2.6. Haloperidol Induced Catalepsy

Haloperidol causes dysfunctioning of various neurotransmitters such as acetylcholine, GABA, and serotonin. Pathology of haloperidol induced catalepsy underlying increased oxidative stress. Haloperidol, an antipsychotic drug, blocks central dopamine receptor in striatum. It also produces a behavioral state in animals like mice and rats in which they fail to correct externally imposed postures (called catalepsy); thus, keeping the above fact in mind, the haloperidol induced catalepsy model was selected. The method described by Elliott and Close in 1990 [20] was followed for the anticataleptic activity. The animals were divided into five groups (*n* = 6). Group I served as vehicle control, Group II served as standard, Levodopa (6 mg/kg, p.o.), and Groups III–V served as test group treated with PEFRE (100, 200, and 400 mg/kg, p.o.), respectively. Standard bar test was used to measure the catalepsy. Catalepsy was induced by haloperidol (1 mg/kg, i.p.) and examined at every 30 min interval for 210 min. The duration for which the rat retains the forepaws extended and resting on the elevated bar was considered as cataleptic score. A cut-off time of 5 min was applied.

### 2.7. Induction of Parkinsonism by 6-OHDA

The rats were anesthetized with an intraperitoneal injection of 50 mg/kg of sodium pentobarbital and were fixed in a stereotaxic apparatus [21, 22]. A stainless steel needle (0.28 mm o.d) was inserted unilaterally into the substantia nigra with the following coordinates: anterior/posterior: −4.8 mm; medial/lateral: −2.2 mm; ventral/dorsal: −7.2 mm–3.5 mm from bregma, and injection of 6-OHDA (20 *μ*g of 6-OHDA hydrobromide in 4 *μ*L 0.9% saline with 0.02 *μ*g/mL ascorbic acid) was then made over 5 min and the needle was left in place for a further 5 min. Then the skull was secured with stainless metallic screws and the wound area was covered by dental cement. Each rat was housed individually following the surgical procedure. Sham operated animals were also treated in the same manner, but they received equivalent volumes of normal saline instead of 6-OHDA.

### 2.8. Experimental Design

Animals were divided into six groups of 6 rats in each group. Group I served as sham operated animals and received normal saline (10 mL/kg, p.o.); Groups II to VI were induced with parkinsonism by 6-OHDA as follows: Group II served as a 6-OHDA control and received normal saline (10 mL/kg), Group III served as a L DOPA (6 mg/kg, p.o.), and Groups IV to VI served as a test drug, PEFRE (100, 200, and 400 mg/kg, p.o., resp.). The treatment of animals was started after 48 h of induction with 6-OHDA according to their respective group once a day for 55 days.

### 2.9. Behavioral Assessment

All behavioral assessment was performed by an observer blinded to the group. Different tests were performed at different time points after lesion.

#### 2.9.1. Locomotor Activity

The spontaneous locomotor activity was monitored using digital actophotometer (Hicon instrument, India) equipped with infrared sensitive photocells. The apparatus was placed in a darkened, light and sound attenuated, and ventilated testing room. Each interruption of a beam on the *x* or *y* axis generated an electric impulse, which was presented on a digital counter. Each animal was observed over a period of 5 min on days 15, 20, 25, 30, 35, 40, 45, 50, and 55 following 6-OHDA administration and values were expressed as counts per 5 min [23].

#### 2.9.2. Rotarod Activity

All animals were evaluated for grip strength by using the rotarod. The rotarod test is widely used in rodents to assess their “minimal neurological deficit” such as motor function and coordination. Each rat was given a prior training session before initialization of therapy to acclimatize them on a rotarod apparatus (EIE instrument, India). Animal was placed on the rotating rod with a diameter of 7 cm (speed 25 rpm). Each rat was subjected to three separate trials at 5 min interval on days 15, 20, 25, 30, 35, 40, 45, 50, and 55 following 6-OHDA administration and cut-off time (180 s) was maintained throughout the experiment. The average results were recorded as fall of time [24].

### 2.10. Dissection and Homogenization

After the treatment period, animals were scarified by decapitation under mild anesthesia. The brains were immediately removed, forebrain was dissected out, and cerebellum was discarded. Brains were put on ice and rinsed in ice-cold isotonic saline to remove blood. A 10% (w/v) tissue homogenate was prepared in 0.1 M phosphate buffer (pH 7.4). The homogenate was centrifuged at 10,000 g for 15 minutes and aliquots of supernatant obtained were used for biochemical estimation.

### 2.11. Biochemical Estimation

#### 2.11.1. Malondialdehyde (MDA) Level

The amount of malondialdehyde was used as an indirect measure of lipid peroxidation and was determined by reaction with thiobarbituric acid (TBA) [25]. Briefly, 1 mL of aliquots of supernatant was placed in test tubes and added to 3 mL of TBA reagent: TBA 0.38% (w/w), 0.25 M hydrochloric acid (HCl), and trichloroacetic acid (TCA 15%). The solution was shaken and placed for 15 min, followed by cooling in an ice bath. After cooling, solution was centrifuged to 3500 g for 10 min. The upper layer was collected and assessed with a spectrophotometer at 532 nm. All determinations were made in triplicate. Results were expressed as nanomoles per mg of protein. The concentration of MDA was calculated using the formula

(2) $$\begin{array}{c} \text{Conc. of MDA}=\frac{{\text{Abs}}_{532}\times 100\times V_{T}}{\left(1.56\times 10^{5}\right)\times W_{T}\times V_{U}}, \end{array}$$

where Abs_532_ is absorbance, *V*_*T*_ is total volume of mixture (4 mL), 1.56 × 10^5^ is molar extinction coefficient, *W*_*T*_ is weight of dissected brain (1 g), and *V*_*U*_ is aliquot volume (1 mL).

#### 2.11.2. Superoxide Dismutase (SOD) Level

SOD activity was determined according to the method described by Beyer and Fridovich in 1987 [26]. 0.1 mL of supernatant was mixed with 0.1 mL EDTA (1 × 10^−4 ^M), 0.5 mL of carbonate buffer (pH 9.7), and 1 mL of epinephrine (1 mM). The optical density of formed adrenochrome was read at 480 nm for 3 min on spectrophotometer. The enzyme activity was expressed in terms of U/min/mg. One unit of enzyme activity is defined as the concentration required for the inhibition of the chromogen production by 50% in one minute under the defined assay conditions.

#### 2.11.3. Catalase (CAT) Level

The catalase activity was assessed by the method of Aebi in 1974 [27]. The assay mixture consists of 0.05 mL of supernatant of tissue homogenate (10%) and 1.95 mL of 50 mM phosphate buffer (pH 7.0) in 3 mL cuvette. 1 mL of 30 mM hydrogen peroxide (H_2_O_2_) was added and changes in absorbance were followed for 30 s at 240 nm at 15 s intervals. The catalase activity was calculated using the millimolar extinction coefficient of H_2_O_2_ (0.071 mmol cm^−1^) and the activity was expressed as micromoles of H_2_O_2_ oxidized per minute per milligram protein:

(3) CAT activity=δO.D.E×Vol.  of  SamplemL×mg of protein,

where *δ*O.D. is change in absorbance/minute; *E* is extinction coefficient of hydrogen peroxide (0.071 mmol cm^−1^).

#### 2.11.4. GSH Level (Reduced Glutathione)

For the estimation of reduced glutathione, the 1 mL of tissue homogenate was precipitated with 1 mL of 10% TCA. To an aliquot of the supernatant, 4 mL of phosphate solution and 0.5 mL of 5,5′-dithiobis-(2-nitrobenzoic acid) (DTNB) reagent were added and absorbance was taken at 412 nm [28]. The values were expressed as nM of reduced glutathione per mg of protein:

(4) $$\begin{array}{c} \text{GSH level}=\frac{Y-0.00314}{0.0314}\times \frac{D_{F}}{B_{T}\times V_{U}}, \end{array}$$

where *Y* is Abs_412_ of tissue homogenate, *D*_*F*_ is dilution factor (1), *B*_*T*_ is brain tissue homogenate (1 mL), and *V*_*U*_ is aliquot volume (1 mL).

### 2.12. Histopathological Studies

The brains from control and experimental groups were fixed with 10% formalin and embedded in paraffin wax and cut into longitudinal section of 5 *μ*m thickness. The sections were stained with hemotoxylin and eosin dye for histopathological observation.

### 2.13. Statistical Analysis

All the values were expressed as mean ± SEM. Statistical evaluation of the data was done by one-way ANOVA (between control and drug treatments) followed by Dunnett's *t*-test for multiple comparisons and two-way ANOVA followed by Bonferroni's multiple comparison test, with the level of significance chosen at *p* < 0.001 using Graph-Pad Prism 5 (San Diego, CA) software.

## 3. Results

### 3.1. Phytochemical Screening

Table 1 showed the phytochemical screening of the different extract of* F. religiosa.*

### 3.2. Acute Toxicity

The PEFRE was found to be safe at all the doses used and there was no mortality found up to the dose of 4000 mg/kg of PEFRE when administered orally. Therefore, we have taken 400 mg/kg as the therapeutic dose and made variations by taking 100 mg/kg as lower dose and 400 mg/kg as higher dose.

### 3.3. The Effects of PEFRE on Haloperidol Induced Catalepsy

**Figure 1:**
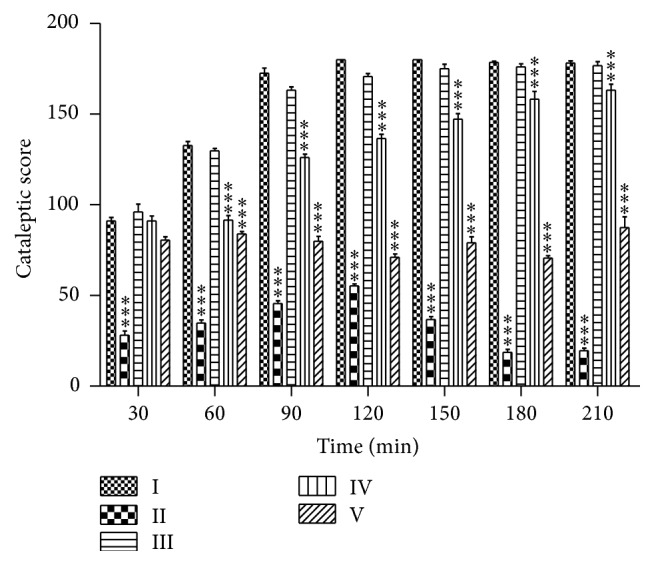
Effect of PEFRE on haloperidol induced catalepsy in rats. Group I: vehicle control group; Group II: Levodopa + haloperidol treated group; Group III: PEFRE (100 mg/kg) + haloperidol treated group; Group IV: PEFRE (200 mg/kg) + haloperidol treated group; Group V: PEFRE (400 mg/kg) + haloperidol treated group. ^*∗∗∗*^ *p* < 0.001 as compared to Vehicle treated control group.

Chronic oral administration of higher doses of PEFRE (200 and 400 mg/kg) showed significant (*p* < 0.001) reduction in cataleptic score from 60 min to 210 min as compared to vehicle treated animals. Administration of PEFRE (100 mg/kg) did not show significant activity. Treatment with Levodopa (6 mg/kg) significantly (*p* < 0.001) reduced duration of catalepsy as compared to vehicle treated group (Figure 1).

### 3.4. The Effects of PEFRE on 6-OHDA Induced Parkinson's Disease

#### 3.4.1. The Effects of PEFRE on 6-OHDA Induced Parkinson's Disease in the Locomotor Activity

**Figure 2:**
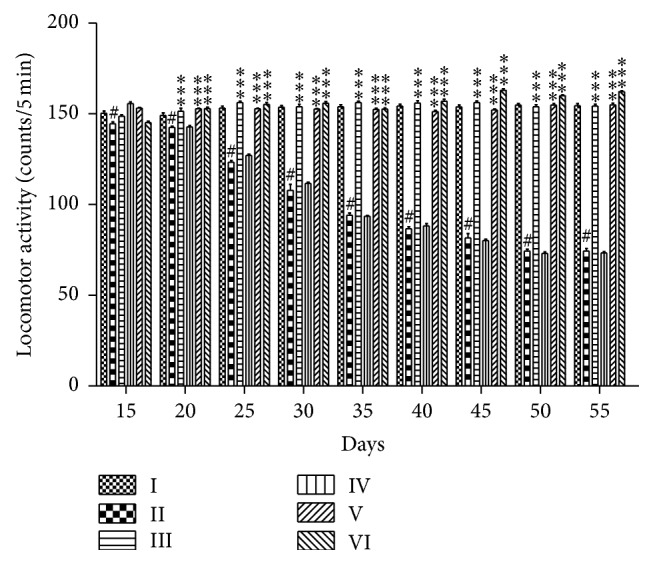
The effects of PEFRE on 6-OHDA induced Parkinson's disease in the Locomotor activity. Group I: Vehicle control group; Group II: 6-OHDA treated group; Group III: Levodopa + 6-OHDA treated group; Group IV: PEFRE (100 mg/kg) + 6-OHDA treated group; Group V: PEFRE (200 mg/kg) + 6-OHDA treated group; Group VI: PEFRE (400 mg/kg) + 6-OHDA treated group. ^*∗∗∗*^ *p* < 0.001 as compared to 6-OHDA treated negative control group (Group II). ^#^ *p* < 0.001 in 6-OHDA treated negative control group (Group II) compared to vehicle treated control group (Group I).

Total locomotor activity of rats in 6-OHDA treated group was significantly (*p* < 0.001) reduced as compared to vehicle treated group. Oral administration of PEFRE of different doses (200 and 400 mg/kg) showed significant (*p* < 0.001) increase in the locomotor activity from day 20 to 55 as compared to 6-OHDA treated control animals. Administration of PEFRE (100 mg/kg) did not show significant activity. Levodopa (6 mg/kg) significantly (*p* < 0.001) increased locomotor activity (Figure 2).

#### 3.4.2. The Effects of PEFRE on 6-OHDA Induced Parkinson's Disease in the Rotarod Performance

**Figure 3:**
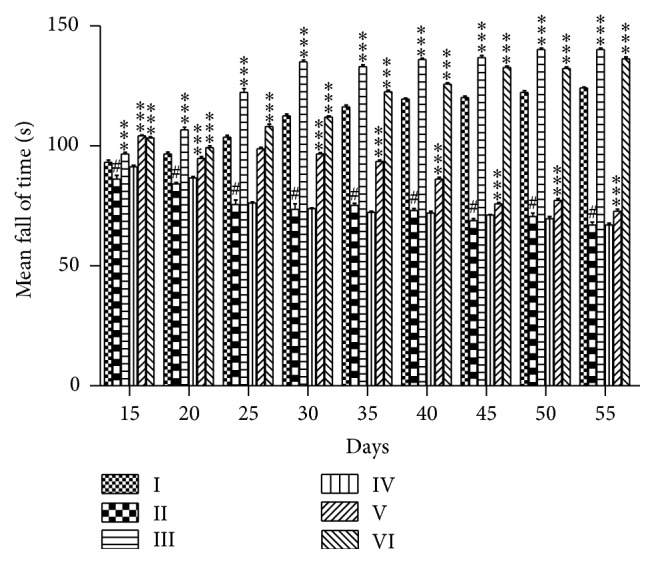
The effects of PEFRE on 6-OHDA induced Parkinson's disease in the rotarod performance. Group I: vehicle control group; Group II: 6-OHDA treated group; Group III: Levodopa + 6-OHDA treated group; Group IV: PEFRE (100 mg/kg) + 6-OHDA treated group; Group V: PEFRE (200 mg/kg) + 6-OHDA treated group; Group VI: PEFRE (400 mg/kg) + 6-OHDA treated group. ^*∗∗∗*^ *p* < 0.001 as compared to 6-OHDA treated negative control group (Group II). ^#^ *p* < 0.001 6-OHDA treated negative control group (Group II) compared to vehicle treated control group (Group I).

Treatment with 6-OHDA significantly decreased the fall of time when compared to the vehicle control animals. Chronic oral administration of PEFRE (200 and 400 mg/kg) significantly (*p* < 0.001) increased the fall of time when compared to 6-OHDA group from day 15 to day 55. Levodopa (6 mg/kg) significantly (*p* < 0.001) increased the fall of time as compared to 6-OHDA group. Administration of PEFRE (100 mg/kg) did not show significant activity (Figure 3).

#### 3.4.3. The Effects of PEFRE on 6-OHDA Induced Parkinson's Disease in MDA, CAT, SOD, and GSH Level

Administration of 6-OHDA resulted in significant changes in biochemical parameters when compared to the vehicle control animals. The inoculation of 6-OHDA induced oxidative stress, as indicated by increased MDA level, and decreased CAT, SOD, and GSH levels when compared to vehicle control animals in brain levels. The treatment with pet. ether extract of FRE (400 mg/kg, p.o.) showed significant (*p* < 0.001) decrease in MDA level compared to OHDA rats. Similarly, daily administration of PEFRE (400 mg/kg) attenuated the increase in SOD and CAT activity with 6-OHDA treated group. Pretreatment with PEFRE (400 mg/kg) significantly (*p* < 0.001) increased GSH levels in the brain as compared to 6-OHDA treated animals, thus preventing the reduction in GSH induced by 6-OHDA (Table 2).

#### 3.4.4. Effect of PEFRE on Histopathological Changes in the Brain of Normal and 6-OHDA Treated Animals

**Figure 4:**
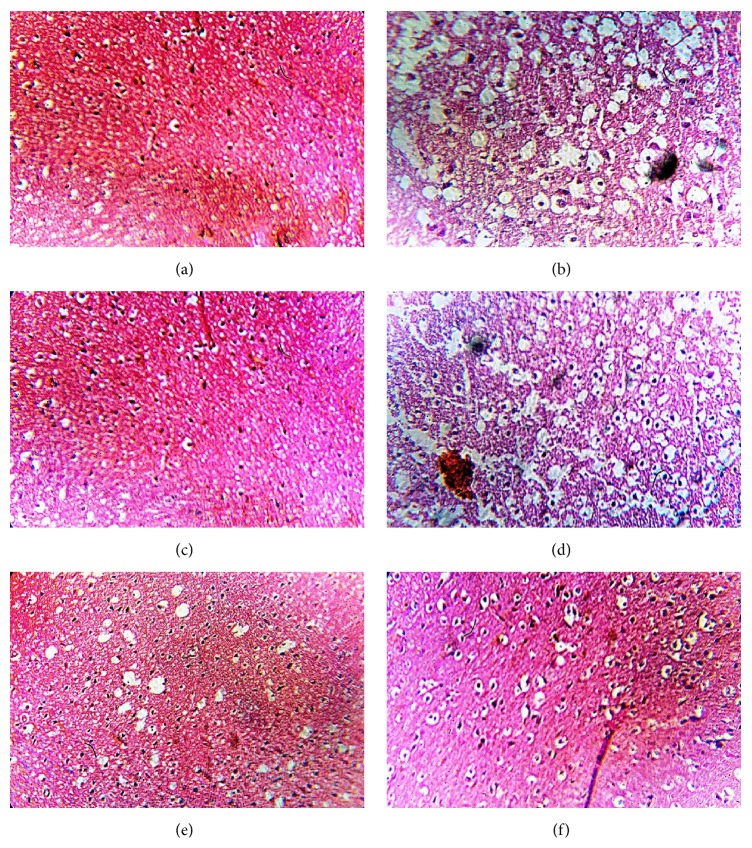
Effect of PEFRE on histopathological changes in the brain of normal and 6-OHDA treated animals (H&E staining; original magnification, 40x). (a) Normal control showing normal brain architecture. (b) Rats treated with 6-OHDA showing degeneration of neurons. (c) Rats treated with 6-OHDA and Levodopa (6 mg/kg) showing minimal changes in neuronal cell integrity and architecture. (d) Rats treated with 6-OHDA and PEFRE (100 mg/kg) showing mild decrease in neurons and cellular hypertrophy. (e) PEFRE (200 mg/kg) and (f) PEFRE (400 mg/kg) treated rats showing minimal changes in neuronal cell populations.

The histopathological study showed that neurotoxins, that is, 6-OHDA, caused marked hypertrophic changes, increased intracellular space, infiltration of neutrophils, decreased density of cells, alterations of architecture, hemorrhage, and neuronal damage and even cell death. Furthermore, many neurons were shrunken, pyknotic, and darkly stained with small nuclei (Figure 4(b)) compared with normal vehicle treated rats (Figure 4(a)). There is significant reversal of neuronal damage or neuronal alterations observed in Levodopa (6 mg/kg) treated rats (Figure 4(c)) and PEFRE treated rats at doses of 200 (Figure 4(e)) and 400 mg/kg (Figure 4(f)). Treatment with PEFRE (100 mg/kg) did not show significant recovery of neuronal damage (Figure 4(d)).

## 4. Discussion

Parkinson's disease is a chronic neurodegenerative disorder characterized by loss of dopamine neurons of the SNpc. The pathogenesis of PD includes oxidative stress, protein accumulation like a-synuclein, mitochondrial dysfunction, apoptosis, and neuronal excitotoxicity. Among all, oxidative stress is a crucial pathological mechanism for PD. SNpc is more vulnerable to reactive oxygen species as it contains more amount of dopamine.

In the present study, we evaluated the effect of pet. ether extract of* F. religiosa* in neurotoxins (haloperidol and 6-OHDA) induced Parkinson disease in experimental animals.

Haloperidol induced catalepsy is a widely accepted animal model of PD. Haloperidol (nonselective D_2_ dopamine antagonists) provides a pharmacological model of parkinsonism by interfering with the storage of catecholamine's intracellularly, resulting in dopamine depletion in nerve endings. In the present study, haloperidol (1 mg/kg, i.p.) induced significant catalepsy in rats as evidenced by a significant increase in the time spent on the block as compared to vehicle treated animals. Treatment with* F. religiosa* significantly reduced the catalepsy in haloperidol treated rats in dose dependent manner. The PEFRE at doses of 200 and 400 mg/kg showed protective effect against haloperidol induced catalepsy indicated that this plant has an ability to protect dopaminergic neurotransmission in striatum.

There are well-known pharmacological PD models in mammalian systems including the classical and highly selective neurotoxin 6-hydroxydopamine (6-OHDA), as well as 1-methyl-4-phenyl-1,2,3,6-tetrahydropyridine (MPTP) and its metabolite, MPP+ (1-methyl-4-phenylpyridinium ion). These toxins cause decreased ATP production, increased ROS production, and increased apoptosis of DAergic cells [29].

The efficacy of* F. religiosa* in 6-OHDA induced PD has not been well established. In the present study, 6-OHDA administration to rats caused a significant decrease in locomotor activity and muscle activity. Lack of motor coordination and maintenance of normal limb posture has been reported in PD condition. The evaluated data suggested damage to the dopaminergic neurons and progression of Parkinson's disease like behavioral abnormalities in rats exposed to 6-OHDA. Pretreatment of rats with PEFRE at the doses of 200 and 400 mg/kg exhibited significant increase in locomotor activity and increase in muscle activity and thus could be proved with possible action on CNS.

Oxidative stress generated as a result of mitochondrial dysfunction particularly mitochondrial complex-1 impairment plays an important role in the pathogenesis of PD. The oxidative stress was measured through determination of levels of malondialdehyde, catalase, superoxide dismutase, and reduced glutathione in the brain tissue.

6-OHDA generates an increase in the production of hydrogen peroxide and free radicals [30, 31]. These reactive oxygen species are generated through the nonenzymatic breakdown of 6-OHDA or direct inhibition of complex-I and complex-IV of the mitochondrial electron transport chain [31–33]. The resulting ROS production from 6-OHDA breakdown leads to lipid peroxidation, protein denaturation, and increases in glutathione, which are found in PD patients [34].

Lipid peroxidation, a sensitive marker of oxidative stress, was estimated by measuring the levels of thiobarbituric acid. Lipid peroxidation occurs due to attack by radicals on double bond of unsaturated fatty acid and arachidonic acid which generate lipid peroxyl radicals and that initiate a chain reaction of further attacks on other unsaturated fatty acid. As we know, lipid peroxidation is the process of oxidative degradation of polyunsaturated fatty acids and its occurrence in biological membranes causes impaired membrane function, impaired structural integrity [35], decreased fluidity, and inactivation of number of membrane bound enzymes. Increased levels of the lipid peroxidation product have been found in the substantia nigra of PD patient. In the present investigation, similar results were observed in the brain homogenate of 6-OHDA treated control animals.

Catalase is an antioxidant which helps in neutralizing the toxic effects of hydrogen peroxide. Hydrogen peroxide is converted by the catalase enzyme to form water and nonreactive oxygen species, thus preventing the accumulation of precursor to free radical biosynthesis. Oxidative stress results in decrease in catalase level. 6-OHDA inoculation in rats induced oxidative stress, as indicated by a decrease in the catalase levels.

Superoxide dismutase (known as SOD) is an enzyme which acts as a catalyst in the process of dismutation of superoxide into nonreactive oxygen species and hydrogen peroxide. It is therefore a critical antioxidant defense which is present in nearly all cells which are exposed to oxygen [36, 37]. Superoxide dismutase helps in neutralizing the toxic effects of free radicals [38, 39]. 6-OHDA treated control group showed a decrease in the level of SOD in the brain of animals, thus indicative of production of oxidative stress.

GSH, potent enzymes, are an important factor in etiology of PD [40]. The depletion of reduced glutathione in the substantia nigra in Parkinson's disease could be the result of neuronal loss. As a matter of fact, the positive correlation has been found to exist between the extent of neuronal loss and depletion of glutathione [41]. A decrease in the availability of reduced glutathione would impair the capacity of neurons to detoxify hydrogen peroxide and increase the risk of free radical formation and lipid peroxidation. A reduction in GSH levels was evident in 6-OHDA treated control animals.

Thus, the 6-OHDA per se group showed a significant increase in the levels of thiobarbituric acid (which is an indication of extent of lipid peroxidation) and decrease in the levels of SOD and GSH in the brain as compared to the vehicle treated control animals. All these indicate an increase in the oxidative stress in the brain of animals treated with 6-OHDA. Pretreatment with higher dose of petroleum ether extract of* F. religiosa* (400 mg/kg) resulted in a decrease in MDA level and increase in the levels of SOD, catalase, and GSH, indicating its antioxidant effect in the brain of 6-OHDA treated animals.

Histopathological findings showed that pet. ether extract of* Ficus religiosa* treated animals had decreased infiltration of neutrophils, reduced intracellular space, increased density of cells, and regained normal architecture and moderate necrosis in striatum region of brain.

## 5. Conclusion

In view of the above facts, we are concluding that petroleum ether extract of* Ficus religiosa* plant showed to be an antioxidant and showed a promising effect in animals with Parkinson's disease. And we appreciate further detailed molecular studies with this drug in anti-Parkinson's pharmacology and toxicology and also characterization of active constituents responsible for neuroprotective effect.

## Figures and Tables

**Table 1:** Phytochemical investigation of *F. religiosa* Linn. leaves.

Sr. number	Name of test	Pet. ether	Ethyl acetate	Ethanol
1	Tannins and phenols	−ve	−ve	+ve
2	Alkaloids	+ve	+ve	+ve
3	Saponins	−ve	−ve	−ve
4	Terpenoids	−ve	+ve	+ve
5	Steroids	−ve	−ve	−ve
6	Flavonoids	−ve	+ve	+ve
7	Carbohydrates	−ve	−ve	−ve
8	Glycosides	−ve	−ve	+ve
9	Phlobatannins	−ve	−ve	−ve
10	Amino acid	−ve	−ve	−ve
11	Protein	−ve	−ve	−ve

**Table 2:** Effect of PEFRE on the levels of lipid peroxidation (MDA), catalase (CAT), superoxide dismutase (SOD), and reduced glutathione (GSH) in the brain of 6-OHDA treated animals.

Group	MDA (nM/mg of protein)	CAT (*μ*moles of H_2_O_2_ used/min/mg protein)	SOD (units/mg protein)	GSH (nM/mg of protein)
Vehicle control	1.311 ± 0.09315	5.788 ± 0.046	3.185 ± 0.1852	4.371 ± 0.07576
6-OHDA control	2.616 ± 0.1602^#^	3.463 ± 0.035^#^	2.06 ± 0.1068^#^	3.985 ± 0.020^#^
Levodopa	1.659 ± 0.03551^*∗∗*^	6.525 ± 0.20^*∗∗∗*^	9.667 ± 0.8333^*∗∗∗*^	7.023 ± 0.6013^*∗∗∗*^
PEFRE (100)	2 ± 0.1558	3.544 ± 0.15	2.419 ± 0.8732	4.182 ± 0.01312
PEFRE (200)	1.975 ± 0.2153	4.234 ± 0.11	2.801 ± 0.5034	4.614 ± 0.1312
PEFRE (400)	1.347 ± 0.2501^*∗∗∗*^	6.22 ± 0.31^*∗∗∗*^	8.833 ± 0.8333^*∗∗∗*^	8.455 ± 0.03936^*∗∗∗*^

Values are expressed as mean ± SEM. ^*∗*^ *p* < 0.05, ^*∗∗*^ *p* < 0.01, and ^*∗∗∗*^ *p* < 0.001 as compared to 6-OHDA treated control group (Group II) [Groups III to VI were compared with Group II], ^#^ *p* < 0.001 as compared to vehicle treated group (Group I) [Group II was compared with Group I].
